# Patient satisfaction with computer-assisted structured initial assessment facilitating patient streaming to emergency departments and primary care practices: results from a cross-sectional observational study accompanying the DEMAND intervention in Germany

**DOI:** 10.1186/s12875-022-01825-5

**Published:** 2022-08-23

**Authors:** Ingmar Schäfer, Agata Menzel, Tobias Herrmann, Jan Hendrik Oltrogge, Dagmar Lühmann, Martin Scherer

**Affiliations:** 1 Department of Primary Medical Care, University Medical Center Hamburg-Eppendorf, Martinistr. 52, 20246 Hamburg, Germany; 2 aQua Institut für angewandte Qualitätsförderung und Forschung im Gesundheitswesen GmbH, Göttingen, Germany

**Keywords:** Emergency care, Patient streaming, Emergency department crowding

## Abstract

**Background:**

Patient numbers in emergency departments are on the rise. The DEMAND intervention aims to improve the efficacy of emergency services by computer-assisted structured initial assessment assigning patients to emergency departments or primary care practices. The aims of our study were to evaluate patient satisfaction with this intervention and to analyse if reduced patient satisfaction is predicted by sociodemographic data, health status or health literacy.

**Methods:**

We conducted a cross-sectional patient survey in emergency departments and co-located primary care practices. Each intervention site was planned to participate for two observation periods, each with a duration of one full week. Study participants were recruited by the local staff. The patients filled out a written questionnaire during their waiting time. Patient satisfaction was assessed by agreement to four statements on a four point Likert scale. Predictors of patient satisfaction were identified by multilevel, multivariable logistic regression models adjusted for random effects at the intervention site level.

**Results:**

The sample included 677 patients from 10 intervention sites. The patients had a mean age of 38.9 years and 59.0% were women. Between 67.5% and 55.0% were fully satisfied with aspects of the intervention. The most criticised aspect was that the staff showed too little interest in the patients’ personal situation. Full satisfaction (“clearly yes” to all items) was reported by 44.2%. Reduced patient satisfaction (at least one item rated as “rather yes”, “rather no”, “clearly no”) was predicted by lower age (odds ratio 0.79 for ten years difference, 95% confidence interval 0.67/0.95, *p* = 0.009), presenting with infections (3.08,1.18/8.05,*p* = 0.022) or injuries (3.46,1.01/11.82,*p* = 0.048), a higher natural logarithm of the symptom duration (1.23,1.07/1.30,*p* = 0.003) and a lower health literacy (0.71 for four points difference, 0.53/0.94,*p* = 0.019).

**Conclusions:**

The patients were for the most part satisfied with the intervention. Assessment procedures should be evaluated a) regarding if all relevant patient-related aspects are included; and whether patient information can be improved b) for patients with strong opinions about cause, consequences and treatment options for their health problem; and c) for patients who have problems in the handling of information relevant to health and healthcare.

**Trial registration:**

German Clinical Trials Register (https://www.drks.de/drks_web/setLocale_EN.do) no. DRKS00017014.

**Supplementary Information:**

The online version contains supplementary material available at 10.1186/s12875-022-01825-5.

## Background

Patient numbers in emergency departments are on the rise while treatment of emergencies by outpatient services is diminishing [1, 2]. Emergency department crowding can be a threat to patient safety. For example, assessment of symptoms and delivery of care can be delayed, the hospital staff is less likely to adhere to clinical practice guidelines and the risk of certain adverse events including mortality might increase [3–5]. Ambulatory care-sensitive conditions, defined as health problems for which hospital stays could be prevented by standard outpatient care [6, 7] are one factor contributing to emergency department crowding. Treatment of ambulatory care-sensitive conditions in emergency departments is also associated with largely increased healthcare costs [8, 9].

A substantial proportion of the patients in emergency departments do not consider their medical condition to be an emergency [10]. The rationale of patients for visiting the emergency departments often differs from a clinician´s perspective and includes perceptions regarding a limited availability of outpatient services, patient preferences and the context in which the health problem occurred [10–12]. Many patients report that they do not know relevant outpatient emergency services they could use as an alternative to the emergency department [10, 11], which might indicate missing information, reduced access to these services or deficits in the patients’ health literacy. Another factor probably influencing the patients’ healthcare use is their satisfaction with medical services. Patient satisfaction describes the quality of healthcare from a patient perspective and is likely to affect patient steering, the amount of time that the patients require from medical and nursing staff and their compliance [13–15].

One possible strategy for reducing crowding in emergency departments is co-location of primary care services in the emergency department [16]. However, recent reviews suggest that evidence regarding effectiveness and safety of patient streaming to primary care services is limited and outdated [17, 18]. The DEMAND intervention addresses this research gap. The intervention aims to improve the efficacy of emergency services by computer-assisted structured initial assessment assigning patients to the emergency services suited best to their health problems, eg, to emergency departments or primary care practices. DEMAND is implemented at the telephone counselling services of the Associations of Statutory Health Insurance Physicians and at emergency departments and co-located primary care practices [2, 19].

The accompanying research to the DEMAND intervention is composed of three subprojects evaluating a) the effects of the intervention on service utilisation; b) the experience of healthcare professionals with implementing the intervention; and c) the patients’ perspective on process and outcome of the intervention including reasons for non-use of recommended services and patient satisfaction [19, 20]. This study was conducted in the context of the third subproject and aimed 1) to evaluate the patient satisfaction with the computer-assisted structured initial assessment at emergency departments and co-located primary care practices and 2) to analyse if reduced patient satisfaction with this intervention is predicted by sociodemographic data, health status or health literacy of the patients.

## Methods

### Design, setting and participants

We conducted a cross-sectional observational study based on a survey of patients from participating hospitals. The DEMAND intervention was scheduled for implementation in 18 emergency departments and co-located primary care practices in seven German federal states. However, five intervention sites did not start regular operations during the observation period. Another two intervention sites included only paediatric emergency services und were therefore excluded. Thus, the accompanying research focused on eleven intervention sites. Participation status of each intervention site can be found in Table A in the additional files.

Before starting the survey, we conducted a pre-test in six intervention sites between 20 May and 28 July 2019. Each site recruited patients for one full week. In total, 107 questionnaires were returned. Subsequent talks with the staff involved in the implementation of the pre-test and the data resulting from the pre-test were used for revising our research methods and questionnaire.

In the main survey, each intervention site was planned to participate in our study for two observation periods, each with a duration of one full week (from Monday to Sunday). The calendar dates of the observation periods were randomly assigned in the time frame between 16 September 2019 and 29 March 2020. However, due to the COVID-19 pandemic and the related lockdown measures, we had to stop data collection after 8 March 2020 and three observation periods could not be realised.

On each day of the weeks selected for data collection, study participants were recruited by the staff working at the intervention sites. The staff included all patients who received a computer-assisted initial assessment and excluded patients who were less than 18 years old, had insufficient German language skills or could not participate in the survey due to functional impairments (eg, insufficient ability to read or write).

All eligible patients received the questionnaire and the written patient information about the study. The patients filled out the questionnaire during their waiting time after being assigned to primary care practice or emergency department. They expressed their consent to study participation by returning the completed questionnaire to the staff at the intervention site. Retrospectively, we excluded patients if their questionnaire had been completed by a third person.

### Intervention

We evaluated the DEMAND-intervention in emergency departments and co-located primary care practices. Before the intervention, there were heterogeneous, non-systematic and unstructured procedures for patient streaming in this setting, eg, in many cases it depended on the experience and personal opinion of the staff if patients presenting their symptoms to the emergency departments were sent to the primary care practice and vice versa. In the DEMAND-intervention, this decision was based on a computer-assisted structured initial assessment.

**Fig. 1:**
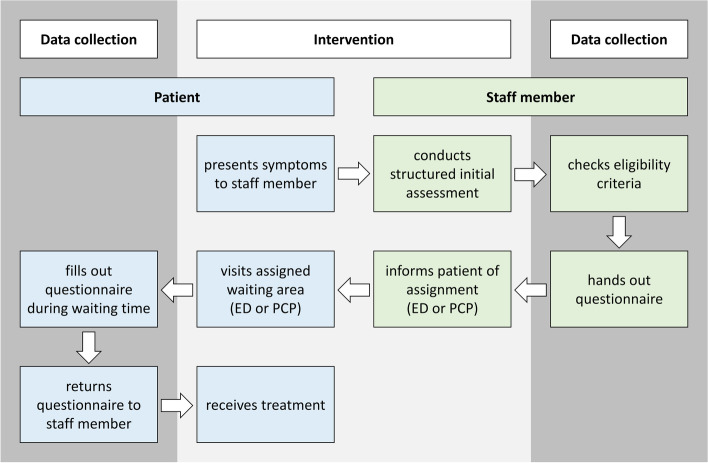
Patient flow regarding intervention and data collection. ED: emergency department; PCP: primary care practice

The patient flow regarding intervention and data collection is shown in Fig. 1. The staff members were instructed to conduct the computer-assisted structured initial assessments with all patients presenting their symptoms at the intervention site. The assessments were facilitated by the software SmED (*“Strukturierte medizinische Ersteinschätzung in Deutschland”* – structured medical initial assessment in Germany), which was developed on the basis of the established SMASS software (“Swiss Medical Assessment System”; https://www.in4medicine.ch/smass.html) adapted to the German healthcare system.

SmED facilitates a structured clinical assessment of treatment urgency considering the 85 most prevalent principal diagnoses in the International Classification of Primary Care (ICPC). Based on a systematic query of symptoms and complaints, previous illnesses and risk factors, SmED assesses the treatment urgency and recommends which service (emergency department or primary care practice) should be utilised. SmED is used as tool supporting decision-making and documentation. The assessment can be ended early if the highest service level is recommended. The staff can deviate from the SmED recommendation in special cases, eg, if services are not available or if in the opinion of the staff a higher treatment urgency is indicated.

### Target and predictor variables

Target variable of our analyses was the patients’ satisfaction with the computer-assisted initial assessment they received. The patients rated their satisfaction with the time and interest that the staff of the intervention site devoted to them during structured initial assessment. They also evaluated if they could say everything they wanted to say and if they would recommend the hospital to friends with acute health problems. The items were assessed on a four point Likert scale (“clearly yes”, “rather yes”, “rather no”, and “clearly no”). The full instrument can be found in the additional files. For our analyses, we dichotomised the answers into the subgroup “full patient satisfaction” consisting of patients rating “clearly yes” on all four items; and the subgroup “reduced patient satisfaction” consisting of patients rating at least one item with “rather yes”, “rather no”, or “clearly no”.

Predictor variables were sociodemographic data, data about the patients’ health status and their self-reported health literacy. Sociodemographic data included age, sex, educational level, migrant status, and the living arrangement (cf. additional files). Living arrangement was dichotomised into patients living alone and patients living together with other people. The migrant status was split into the groups “natives” (ie, if the patients and their parents were born in Germany); “people with migration background” (ie, if the patients were born in Germany, but at least one of their parents were born abroad); and “migrants” (ie, if the patients were born abroad).

The educational level was based on the patients ‘ general and vocational education and coded according to the Comparative Analysis of Social Mobility in Industrial Nations (CASMIN) classification system into the groups “low educational level” for inadequately completed general education, general elementary education or basic vocational qualification; “medium educational level” for secondary school certificate or A level equivalent; and “high educational level” for higher or lower tertiary education [21].

The health status was assessed by the subjective treatment urgency, the duration of symptoms, and the consultation reasons (cf. additional files). Additionally, we measured the health-related quality of life. The subjective treatment urgency was rated on a numerical rating scale ranging from 0 (indicating ‘no urgent need for treatment’) to 10 (indicating ‘very urgent, life threatening’). For our descriptive analyses, the duration of symptoms was coded into seven categories (“less than six hours”; “six hours to less than one day”;”one day to less than three days”; “three days to less than one week”; “one week to less than one month”; “one month to less than one year”; and “one year or more”). For our multivariable models, we used the natural logarithm of the symptom duration.

The consultation reasons were assessed by open questions and retrospectively coded by the project staff (JHO, AM) in the International Classification of Primary Care, Second Revision (ICPC-2) [22], which facilitates grouping by organ systems (eg, “respiratory system” or “psychological disorders”) and by diagnosis type (ie, “symptoms/complaints”, “infections”, “injuries”, “congenital anomalies”, “neoplasms” and “other diagnoses”). Health-related quality of life was assessed by the five level version of the EuroQol Five-Dimension Scale (EQ-5D-5L) comprising the domains mobility, self-care, usual activities, pain or discomfort and anxiety or depression [23]. An EQ-5D summary score was calculated using the German value set. It indicates the value 1.000 for full health, which is reduced by up to five subtrahends between -0.026 and -0.612 depending on the severity of limitations in each of the five dimensions [24].

We measured the self-reported health literacy by the short form of the European Health Literacy Questionnaire (HLS-EU-Q16). It includes 16 questions focusing on the four dimensions accessing, understanding, appraising and applying information to take decisions concerning health care (7 questions), disease prevention (5 questions) and health promotion (4 questions) [25]. The items were rated on a four point Likert scale and dichotomised for our analyses. For each item, we grouped “fairly easy” and “very easy” to the value of 1 and “fairly difficult” and “very difficult” to the value of 0. Thus, the HLS-EU-Q16 summary score ranges from 0 to 16 points. The summary score can be divided in three categories. “Inadequate” health literacy is assumed at 0–8 points, “problematic” health literacy at 9–12 points and “sufficient” health literacy at 13–16 points [26].

### Statistical analyses

The patient satisfaction with the computer-assisted initial assessment streaming patients to emergency departments or co-located primary care practices was analysed by descriptive analyses. The possible predictors of reduced patient satisfaction were analysed in two steps. First, we performed chi-squared-tests and t-tests to describe the differences in sociodemographic data, health status and self-reported health literacy between patients with full and impaired patient satisfaction.

Second, we conducted multilevel, multivariable logistic regression models adjusted for random effects at the intervention site level to analyse the association between predictor variables and reduced patient satisfaction (dependent variable). The potential predictors of reduced patient satisfaction included sociodemographic data, health status, and self-reported health literacy. Results from inferential statistics were reported as ß-coefficients with 95% confidence intervals. Additionally, odds ratios of significant predictor variables were calculated. An alpha level of 5% (*p* < 0.05) was defined as statistically significant. All statistical analyses were performed using Stata 15.1.

## Results

During the observation time, nine intervention sites could be observed for two weeks and one intervention site for one week. One intervention site had to be excluded, because both observation periods had been randomised to the time during the COVID-19 pandemic and therefore no data collection could be realised. The periods of data collection in each intervention site and the local response rates can be found in Table B in the additional files.

**Fig. 2:**
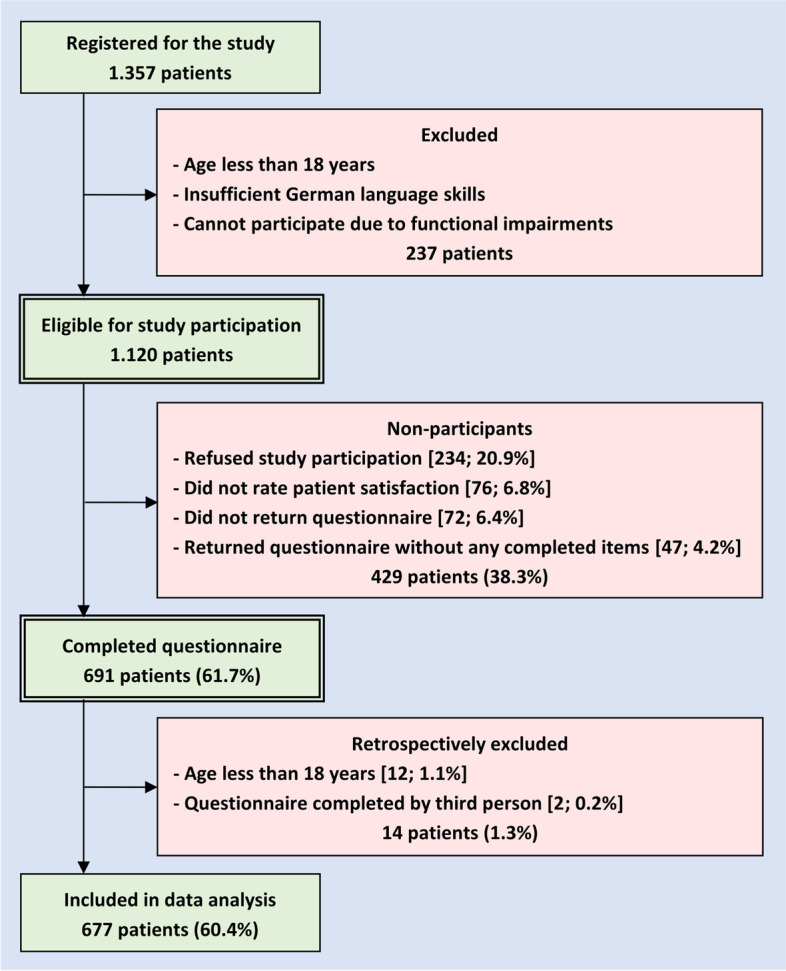
Patient recruitment

The recruitment of patients is described in Fig. 2. In total 1,357 patients were registered for the study. Of these, 237 had to be excluded due to being minors, insufficient German language skills or functional impairments. Out of 1,120 eligible patients 429 did not participate because they refused study participation, did not return the questionnaire or did not complete all relevant items. A completed questionnaire was returned by 691 patients corresponding to a response rate of 61.7%. Retrospectively, we had to exclude 14 patients, because they were less than 18 years old or because they documented in the open-ended questions that the questionnaire was completed by a third person. In the end, 677 patients from 10 intervention sites were included in our data analysis.

Descriptive data of the sample are shown in Table 1. The patients had a mean age of 38.9 years and 59.0% were women. About one quarter of the patients (23.1%) were living alone and more than half of the population (54.4%) had a medium education. Four out of ten patients (39.1%) were migrants or had a migration background. On average, the patients rated their treatment urgency in the medium range (5.7 ± 2.1 points). For 36.3% patients, symptoms had already persisted for three days or more before seeking medical help at the hospital. On average, most patients had mildly to moderately impaired health-related quality of life (0.73 ± 0.26 points) and problematic to sufficient health literacy (11.9 ± 3.3 points).

**Table 1:** Descriptive data of the sample

	**Total** **(*n* = 677)**	**Reduced patient satisfaction** **(*n* = 378)**	**Full patient satisfaction** **(*n* = 299)**	***P***
***Age (in years)***	***38.9* ± *16.0*** ***(n* = *656)***	***37.2* ± *15.4*** ***(n* = *364)***	***41.0* ± *16.4*** ***(n* = *292)***	***0.002***
Sex:				
- men	40.4%	42.2%	38.2%	0.288
- women	59.0%	57.5%	60.8%	
- non-binary	0.6% (*n* = 668)	0.3% (*n* = 372)	1.0% (*n* = 296)	
Living alone	23.1% (*n* = 663)	24.4% (*n* = 369)	21.4% (*n* = 294)	0.369
Educational level (pursuant to CASMIN):				
- low	20.3%	18.6%	22.2%	0.465
- medium	54.4%	54.9%	53.9%	
- high	25.3% (*n* = 612)	26.6% (*n* = 339)	23.8% (*n* = 273)	
Migration status^a^:				
- natives	60.9%	60.0%	62.0%	0.195
- people with migration background	19.7%	22.0%	16.8%	
- migrants	19.4% (*n* = 660)	17.9% (*n* = 368)	21.2% (*n* = 292)	
Subjective treatment urgency	5.7 ± 2.1 (*n* = 577)	5.6 ± 2.1 (*n* = 325)	5.8 ± 2.1 (*n* = 252)	0.220
Duration of symptoms:				
- less than six hours	11.1%	9.5%	13.1%	0.435
- six hours to less than one day	23.4%	22.0%	25.2%	
- one day to less than three days	29.2%	31.5%	26.3%	
- three days to less than one week	17.5%	16.7%	18.6%	
- one week to less than one month	14.2%	15.0%	13.1%	
- one month to less than one year	3.5%	4.2%	2.6%	
- one year or more	1.1% (*n* = 633)	1.1% (*n* = 359)	1.1% (*n* = 274)	
Health-related quality of life (pursuant to EQ-5D-5L, German value set)	0.73 ± 0.26 (*n* = 526)	0.74 ± 0.25 (*n* = 293)	0.73 ± 0.26 (*n* = 233)	0.968
Health literacy (pursuant to HLS-Q16-EU)	11.9 ± 3.3 (*n* = 564)	11.8 ± 3.3 (*n* = 324)	12.1 ± 3.3 (*n* = 240)	0.244

^a^ Natives: patients and their parents were born in Germany; migration background: patients were born in Germany, at least one of their parents were born abroad; migrants: patients were born abroad

Details on the health problems of the patients are shown in Table C in the additional files. The most frequently affected organ systems were the respiratory system (22.0%), the digestive system (19.8%) and the musculoskeletal system (19.6%). The most prevalent health problems were “throat symptom/complaint” (7.9%), “fever” (7.8%) and “headache” (7.5%). Generally, the ICPC-2 diagnosis type “symptoms and complaints” (77.0%) was much more often used than “infections” (17.8%), “injuries” (7.5%) and “other diagnoses” (6.5%).

**Fig. 3:**
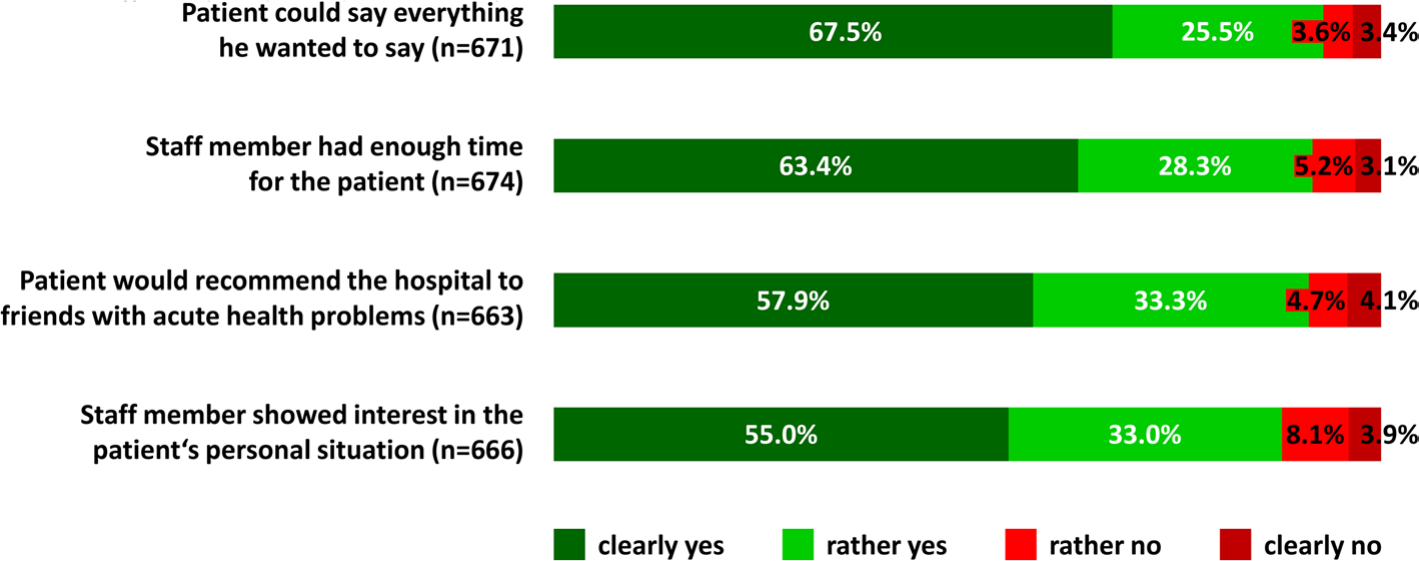
Patient satisfaction

Patient satisfaction with the computer-assisted initial assessment is described in Fig. 3. Depending on the respective item, full agreement (“clearly yes”) ranged between 67.5% and 55.0%. Another 33.3% to 25.5% did rather agree (“rather yes”). Non-agreement was comparably rare and ranged between 8.1% and 3.6% (“rather no”) and 4.1% and 3.1% (“clearly no”), respectively. The item “patient could say everything he wanted to say” received the highest rating (93.0% “clearly yes” or “rather yes”) and the item “staff member showed interest in the patient’s personal situation” the lowest (88.0% “clearly yes” or “rather yes”). Full patient satisfaction (ie, “clearly yes” in all four items) was rated by 44.2% of the patients.

Univariable differences between patients with reduced and full satisfaction are shown in Table 1 and Table C in the additional files. Patients with reduced satisfaction were younger (37.2 ± 15.4 years vs. 41.0 ± 16.4 years, *p* = 0.002) and they had a lower proportion of health problems from the urological system (7.5% vs. 13.0%), a lower prevalence of “dysuria/painful urination” (1.2% vs. 4.1%), and a higher prevalence of “abdominal pain localized other” (8.7% vs. 3.7%). There were no significant differences in other analysed variables including the frequency of the four ICPC-2 diagnosis types found in the data set.

The results from the multivariable models analysing the association of reduced patient satisfaction with sociodemographic data, health status and health literacy are shown in Table 2. A lower age was associated with reduced patient satisfaction (Odds Ratio [OR] 0.79 for ten years difference, 95% confidence interval [CI] 0.67/0.95, *p* = 0.009). Additionally, presenting with infections (OR 3.08, 95% CI 1.18/8.05, *p* = 0.022) or injuries (OR 3.46, 95% CI 1.01/11.83, *p* = 0.048) and a higher duration of symptoms (OR 1.23 for one step on the natural logarithmic scale, 95% CI 1.07/1.40, *p* = 0.003) were related to reduced patient satisfaction. For example, one step on the natural logarithmic scale represents the approximated difference between one and three days, between one and three weeks or between one and three months. We also identified lower health literacy (OR 0.71 for four points difference, 95% CI 0.53/0.94, *p* = 0.019) as predictor of reduced patient satisfaction.

**Table 2:** Predictors of reduced patient satisfaction: Results from logistic regression in mixed models adjusted for random effects on the intervention site level

	**ß (95% CI)**	**odds ratio (95% CI)**	***P***
***Age (per 10 years difference)***	***-0.23 (-0.40/-0.06)***	***0.79 (0.67/0.95)***	***0.009***
Sex^a^: female vs. male	-0.26 (-0.73/0.22)	0.77 (0.48/1.24)	0.288
Living alone	0.003 (-0.55/0.55)	1.00 (0.58/1.74)	0.990
Educational level (pursuant to CASMIN):			
- medium vs. low	0.19 (-0.51/0.89)	1.21 (0.60/2.44)	0.594
- high vs. low	0.29 (-0.47/1.05)	1.34 (0.63/2.86)	0.449
Migration status^b^:			
- people with migration background vs natives	0.39 (-0.23/1.01)	1.48 (0.79/2.76)	0.219
- migrants vs. natives	0.08 (-0.56/0.73)	1.09 (0.57/2.07)	0.801
Subjective treatment urgency	-0.08 (-0.20/0.04)	0.92 (0.82/1.04)	0.196
***Duration of symptoms (in hours: natural logarithm)***	***0.20 (0.07/0.34)***	***1.23 (1.07/1.40)***	***0.003***
Health problems (ICPC-2 chapters):			
- general and unspecified (A)	-0.51 (-1.19/0.16)	0.60 (0.30/1.18)	0.137
- digestive system (D)	0.25 (-0.47/0.97)	1.29 (0.62/2.65)	0.495
- ear (H)	0.25 (-0.88/1.38)	1.29 (0.42/3.99)	0.661
- musculoskeletal system (L)	0.00006 (-0.80/0.80)	1.00 (0.45/2.21)	> 0.999
- neurological system (N)	-0.66 (-1.41/0.10)	0.52 (0.24/1.11)	0.091
- respiratory system (R)	-0.31 (-0.97/0.35)	0.73 (0.38/1.42)	0.358
- skin (S)	-0.51 (-1.34/0.32)	0.60 (0.26/1.38)	0.231
- urological system (U)	-0.70 (-1.65/0.25)	0.50 (0.19/1.29)	0.151
***Health problems (ICPC-2 diagnosis types)***			
- symptoms and complaints	0.28 (-0.70/1.27)	1.33 (0.49/3.57)	0.573
***- infections***	***1.12 (0.16/2.09)***	***3.08 (1.18/8.05)***	***0.022***
***- injuries***	***1.24 (0.009/2.47)***	***3.46 (1.01/11.83)***	***0.048***
- other diagnoses	0.43 (-0.62/1.48)	1.54 (0.54/4.40)	0.421
Health-related quality of life (pursuant to EQ-5D-5L, value set UK)	-0.20 (-1.29/0.88)	0.81 (0.28/2.41)	0.711
***Health literacy (pursuant to HLS-Q16-EU; per 4 points difference)***	***-0.35 (-0.63/-0.06)***	***0.71 (0.53/0.94)***	***0.019***

^a^ Non-binary not compared to other sexes due to low number of cases (*n* = 4) in this subgroup

^b^ Natives: patients and their parents were born in Germany; migration background: patients were born in Germany, at least one of their parents were born abroad; migrants: patients were born abroad; CI: confidence interval

## Discussion

### Statement of principal findings

Almost half of the study participants were fully satisfied with the computer-assisted initial assessment in the DEMAND intervention. Dissatisfaction was comparably rare. In our analyses, satisfaction with the initial assessment was independent of the patients’ sex, educational level, migrant status and living arrangement. Younger patients, however, more often showed a reduced satisfaction than older patients. The severity of the health problems as indicated by impairment in the health-related quality of life was not related to the level of satisfaction, but patients with infections, injuries or conditions that already existed for a long time were less likely to be fully satisfied. Additionally, lower health-literacy was associated with a higher probability of reduced satisfaction with the initial assessment.

### Comparison with the literature

Our population had a mean age of 39 years and 59% were women. An insurance claims data based analysis of the age distribution in outpatient emergency patients showed a comparable mean and variance of age as in our study [27]. However, in two studies observing patients with unselected, non-urgent conditions, the distribution of sex was more balanced than in our study [10, 28].

Between 88 and 93% of the patients in our study were fully or rather satisfied with different aspects of our interventions. The high levels of satisfaction we observed were also found in other studies evaluating different methods of triage in the emergency department, eg, nurse-led triage [29–31]. In a systematic review, determinants of patient satisfaction with nurse-led triage included the nurses’ ability to provide patient-centred care, communication skills, concern for the patient and competence in diagnosing [32]. Many studies highlighted waiting time, particularly in the waiting room, as highly predictive of reduced patient satisfaction [31–36].

We observed that lower age was associated with reduced patient satisfaction. Other socio-demographic factors were not related to the level of satisfaction. With few exceptions [37], most other studies found the same age-dependency of patient satisfaction as our study [38–41]. The results reported in various studies concerning the influence of sex on patient satisfaction are contradictory [38, 39, 42]. Some studies found a higher patient satisfaction in men [41], some in women [43].

Grouped into three categories, 50% of our population had sufficient, 32.8% had problematic, and 17.0% had inadequate health literacy. The rates of problematic and inadequate health literacy were higher than in the general population [44], but comparable to another study in the non-urgent emergency department setting [28]. We observed that a lower health literacy was associated with reduced patient satisfaction. Other studies from different settings and countries found comparable results [45–48].

In contrast to our results, many studies also found that a lower educational level was related to reduced satisfaction [38–40]. As lower educational level is associated with lower health literacy [44, 49, 50], the differences to other studies might therefore be explained by our multivariable models adjusting both factors for each other.

In our study, certain types of health problems were associated with reduced satisfaction, but severity and treatment urgency of the health problems did not predict patient satisfaction. Some other studies also found a relationship between health status and patient satisfaction, eg, a reduced satisfaction in patients with multiple chronic conditions, moderate or severe migraine-related disability or poorly controlled diabetes [38, 51–53]. Higher treatment urgency predicted higher patient satisfaction in some studies [54, 55], while others found no effect [34].

Taken altogether, the results of our study are mostly in line with existing evidence. The dependency of patient satisfaction on factors like age or health literacy might therefore be based to a certain amount on general tendencies in patient satisfaction rather than on than specific problems with our intervention. Nevertheless, achieving sufficient patient satisfaction with the provided services remains an important aim of our intervention.

### Implications for clinical practice

The patients in our study were – for the most part and across most sociodemographic groups – satisfied with the services they received. Only few patients felt that the staff devoted too little time to them or that they could not say everything they wanted to say. The most criticised aspect was that the staff showed too little interest in the patients’ personal situation. Therefore, the staff should be trained to pay more attention to the specific situation of the patients. It also seems important to conduct a continuous monitoring if all relevant aspects for triaging patients appropriately are included in the initial assessments.

The type of health problem that the patients presented was related to their satisfaction. This could reflect associations of the medical care with patient satisfaction [38, 51–53], but it could also be related to our intervention. Patients visiting the emergency department with infections, injuries and long-lasting conditions probably have had a stronger opinions regarding cause, consequences and treatment options for their health problem than patients who come with new and undiagnosed complaints like fever, cough, nausea, or vertigo and they might therefore have had less understanding for the first assessment they received. As waiting times are one of the most important predictors for reduced patient satisfaction [31–36], this patient group should be informed why first assessment is necessary in their specific case.

We also identified that a lower health literacy was associated with reduced satisfaction, which is know from other studies as well [45–48]. With regard to our specific intervention, it could be that patients who have problems in the handling of information relevant to health and healthcare did not understand the rationale and importance of the initial assessment very well. As targeted information can improve patient satisfaction in the inpatient emergency setting [32, 33], it could be helpful to accompany the initial assessment by patient-centred information in easy language explaining why the initial assessment is being conducted and what the patients can expect.

### Strengths and limitations of the study

Unfortunately, it was not possible to conduct a randomised-controlled trial evaluating the patient satisfaction with the computer-assisted initial assessment. For this reason, the satisfaction of patients receiving the intervention could not be compared to patients receiving care as usual. Furthermore, there was no pre-intervention study. We therefore do not know if the low levels of dissatisfaction we observed are related to a change to the better, no change at all or a change to the worse compared to care as usual. But we were able to identify predictors of reduced satisfaction in order to determine if specific patient groups feel dissatisfied with the initial assessment. In these analyses, we used strict criteria for full satisfaction in order to compensate for the missing control group in our study.

However, our definition of full satisfaction, including only patients who “fully agree” to each of the statements, might be too strict for patients who tend to make cautious ratings. In a sensitivity analysis, we therefore adjusted for rating “rather yes” on all items of patient satisfaction, which applied to 47 patients. In this analysis, injuries and infections lost their statistical significance, but lower age (OR 0.80 for ten years difference, 95% CI 0.67–0.97, *p* = 0.021), higher symptom duration (OR 1.22 for one step on the natural logarithmic scale, 95% CI 1.05–1.41, *p* = 0.009) and lower health literacy (OR 0.65 for four points difference, 95% CI 0.48–0.89, *p* = 0.007) were still associated with reduced patient satisfaction.

It must be noted that we did not make qualitative exploration of the patient perspective on our intervention. For this reason, we do not know if it was clear for patients which processes were part of the DEMAND intervention and which related to standard operating procedures of the hospital. The four items assessing patient satisfaction therefore also covered a broader subject and cannot be fully attributed to our intervention. The questions were piloted in a pre-test, but we did not conduct psychometric validation of these items. In our multivariable models, we did not include waiting time and the outcome of the initial assessment as predictor variables. However, both variables might influence patient satisfaction and the set of predictors could therefore be incomplete.

Health literacy was measured by the validated [56] and established [57] questionnaire HLS-EU-Q16. The questionnaire was used in the national GEDA cohort providing representative data on the distribution of health literacy in the general population in Germany [44]. However, the HLS-EU-Q16 was also criticised for using subjective estimates of the patients’ own competencies and for errors regarding the assessment of patient leaflets and screening examinations [58].

Reduced health literacy is known to be related to low socioeconomic status, migration background and language-related communication barriers [59–61]. The statistical analyses were adjusted for educational level and migration status. However, the association between reduced health literacy and low patient satisfaction might still be explained in part by factors like income-related social deprivation, language problems, or cultural factors.

Other limitations of our study include the mode of data collection, the patient selection and the sample size. The patients completed the survey during their waiting time and we do not know how the level of satisfaction with the intervention altered following the completion of their episode of care. As in most surveys, we cannot rule out that recall problems, errors or social desirability have biased the data.

We do not know to what extent the included patients are representative of the studied population. In this context it should be noted that the proportion of women in our sample was higher than in comparable studies. We excluded minors, patients with specific functional limitations and patients, who cannot read or write in German. Additionally, 38.3% of the eligible patients did not participate in our study. We also did not conduct a sample size calculation. It is therefore possible that significant predictors of reduced patient satisfaction were missed due to limited statistical power.

Strengths of our study relate to the selection of observation periods, the standardisation of data collection, the selection of study regions and the statistical methods. The specific calendar dates on which the specific intervention sites were observed had been randomised. The likeliness of bias introduced by events such as flu epidemics or TV reports is therefore reduced. The intervention sites used standardised procedures of data collection and were closely monitored by the scientific staff in the project. The study includes patients from urban and rural areas and many different regions in Germany are covered. And the statistical methods consider potential confounders and the clustering of patients in the specific intervention sites.

## Conclusions

The patients were for the most part satisfied with the intervention. Reduced patient satisfaction was predicted by lower age, presenting with infections or injuries, a higher symptom duration and a lower health literacy. Assessment procedures should therefore be evaluated a) regarding if all relevant patient-related aspects are included; and whether patient information can be improved b) for patients with strong opinions about cause, consequences and treatment options for their health problem; and c) for patients who have problems in the handling of information relevant to health and healthcare.

## Supplementary Information

**Additional file 1: Table A.** Intervention sites, federal states and participation status. **Table B.** Location of intervention sites, periods of data collection and local response rates. **Table C.** Health problems with prevalence ≥ 2% (pursuant to ICPC-2).

**Additional file 2.** Patient questionnaire.

## Data Availability

The datasets generated and analysed during the current study are not publicly available, because the ethics approval does not allowed data sharing, but available from the corresponding author on reasonable request.
