# Supplementary material for: Patient satisfaction with computer-assisted structured initial assessment facilitating patient streaming to emergency departments and primary care practices: results from a cross-sectional observational study accompanying the DEMAND intervention in Germany
Source: BMC Prim Care. 2022 Aug 23;23:213. doi: 10.1186/s12875-022-01825-5 (PMC9397153; doi:10.1186/s12875-022-01825-5)
Supplement: Supplementary file 1 — Additional file 1: Table A. Intervention sites, federal states and participation status. Table B. Location of intervention sites, periods of data collection and local response rates. Table C. Health problems with prevalence ≥ 2% (pursuant to ICPC-2). [file 12875_2022_1825_MOESM1_ESM.docx]

**Table A: Intervention sites, federal states and participation status**

| **City** | **Federal state** | **Participation status** |
| --- | --- | --- |
| Augsburg | Bavaria | Excluded: did not start regular operations |
| Bernau bei Berlin | Brandenburg | Excluded: no data collection possible |
| Bremen | Bremen | Participated in main survey and pre-test |
| Coesfeld | North Rhine-Westphalia | Excluded: only paediatric emergencies |
| Gelsenkirchen | North Rhine-Westphalia | Participated in main survey and pre-test |
| Grevenbroich | North Rhine-Westphalia | Participated in main survey |
| Kiel | Schleswig-Holstein | Participated in main survey |
| Lippstadt | North Rhine-Westphalia | Participated in main survey and pre-test |
| Lübeck | Schleswig-Holstein | Participated in main survey |
| Mainz | Rhineland-Palatinate | Participated in main survey and pre-test |
| Mülheim an der Ruhr | North Rhine-Westphalia | Excluded: did not start regular operations |
| Münster | North Rhine-Westphalia | Participated in main survey and pre-test |
| Neuss | North Rhine-Westphalia | Excluded: did not start regular operations |
| Nürnberg | Bavaria | Excluded: did not start regular operations |
| Reutlingen | Baden-Württemberg | Excluded: only paediatric emergencies |
| Sindelfingen | Baden-Württemberg | Participated in main survey and pre-test |
| Villingen-Schwenningen | Baden-Württemberg | Participated in main survey |
| Würselen | North Rhine-Westphalia | Excluded: did not start regular operations |

**Table B: Location of intervention sites, periods of data collection and local response rates**

| **City** | **Federal state** | **Week** | **Registered** | **Analysed** |
| --- | --- | --- | --- | --- |
| Münster | North Rhine-Westphalia | 16./22.09.2019 | 33 | 8 |
| Lübeck | Schleswig-Holstein | 7./13.10.2019 | 50 | 45 |
| Sindelfingen | Baden-Württemberg | 7./13.10.2019 | 98 | 64 |
| Münster | North Rhine-Westphalia | 14./20.10.2019 | 23 | 18 |
| Mainz | Rhineland-Palatinate | 21./27.10.2019 | 61 | 18 |
| Gelsenkirchen | North Rhine-Westphalia | 28.10./3.11.2019 | 38 | 15 |
| Lippstadt | North Rhine-Westphalia | 28.10./3.11.2019 | 7 | 5 |
| Bremen | Bremen | 4./10.11.2019 | 336 | 182 |
| Villingen-Schwenningen | Baden-Württemberg | 4./10.11.2019 | 88 | 35 |
| Lippstadt | North Rhine-Westphalia | 18./24.11.2019 | 14 | 9 |
| Kiel | Schleswig-Holstein | 16./22.12.2019 | 50 | 28 |
| Gelsenkirchen | North Rhine-Westphalia | 6./12.1.2020 | 49 | 13 |
| Villingen-Schwenningen | Baden-Württemberg | 13./19.1.2020 | 62 | 23 |
| Kiel | Schleswig-Holstein | 20./26.1.2020 | 51 | 25 |
| Grevenbroich | North Rhine-Westphalia | 27.1./2.1.2020 | 5 | 3 |
| Mainz | Rhineland-Palatinate | 27.1./2.1.2020 | 99 | 30 |
| Bremen | Bremen | 3./9.2.2020 | 231 | 120 |
| Sindelfingen | Baden-Württemberg | 10./16.2.2020 | 55 | 33 |
| Grevenbroich | North Rhine-Westphalia | 2./8.3.2020 | 7 | 3 |

**Table C: Health problems with prevalence ≥ 2% (pursuant to ICPC-2)**

|  | **Total (n=617)** | **Reduced patient satisfaction (n=347)** | **Full patient satisfaction (n=270)** | **p** |
| --- | --- | --- | --- | --- |
| Respiratory system (R), thereof: | 22.0% | 23.3% | 20.4% | 0.377 |
| -- Throat symptom/complaint (R21) | 7.9% | 8.4% | 7.4% | 0.665 |
| -- Cough (R05) | 6.0% | 5.8% | 6.3% | 0.782 |
| -- Shortness of breath/dyspnoea (R02) | 3.1% | 2.6% | 3.7% | 0.428 |
| -- Tonsillitis acute (R76) | 2.6% | 2.3% | 3.0% | 0.610 |
| Digestive system (D), thereof: | 19.8% | 21.9% | 17.0% | 0.132 |
| ***-- Abdominal pain localized other (D06)*** | ***6.5%*** | ***8.7%*** | ***3.7%*** | ***0.013*** |
| -- Nausea (D09) | 4.2% | 4.6% | 3.7% | 0.578 |
| -- Vomiting (D10) | 2.9% | 3.8% | 1.9% | 0.165 |
| -- Abdominal pain epigastric (D02) | 2.4% | 2.6% | 2.2% | 0.766 |
| -- Diarrhoea (D11) | 2.1% | 2.3% | 1.9% | 0.697 |
| Musculoskeletal system (L), thereof: | 19.6% | 20.5% | 18.5% | 0.547 |
| -- Back symptom/complaint (L02) | 4.9% | 5.5% | 4.1% | 0.422 |
| -- Foot/toe symptom/complaint (L17) | 2.6% | 2.6% | 2.6% | 0.999 |
| -- Chest symptom/complaint (L04) | 2.3% | 2.3% | 2.2% | 0.945 |
| General and unspecified (A), thereof: | 19.0% | 17.3% | 21.1% | 0.230 |
| -- Fever (A03) | 7.8% | 7.5% | 8.2% | 0.763 |
| -- Chest pain not otherwise specified (A11) | 3.6% | 3.8% | 3.3% | 0.784 |
| -- Pain general/multiple sites (A01) | 3.4% | 2.9% | 4.1% | 0.418 |
| -- Weakness/tiredness general (A04) | 2.4% | 2.3% | 2.6% | 0.818 |
| Neurological system (N), thereof: | 11.5% | 11.5% | 11.5% | 0.986 |
| -- Headache (N01) | 7.5% | 7.2% | 7.8% | 0.788 |
| -- Vertigo/dizziness (N17) | 4.4% | 4.9% | 3.7% | 0.471 |
| Skin (S), thereof: | 12.8% | 13.3% | 12.2% | 0.703 |
| -- Rash localized (S06) | 2.3% | 2.0% | 2.6% | 0.634 |
| ***Urological system (U), thereof:*** | ***9.9%*** | ***7.5%*** | ***13.0%*** | ***0.024*** |
| -- Cystitis/urinary infection other (U71) | 5.0% | 4.6% | 5.6% | 0.594 |
| ***-- Dysuria/painful urination (U01)*** | ***2.4%*** | ***1.2%*** | ***4.1%*** | ***0.019*** |
| Ear (H), thereof: | 5.4% | 5.2% | 5.6% | 0.840 |
| -- Ear pain/earache (H01) | 3.4% | 3.2% | 3.7% | 0.717 |
| Eye (F) | 4.9% | 4.0% | 5.9% | 0.279 |
| Cardiovascular system (K) | 4.9% | 5.2% | 4.4% | 0.670 |
