# Supplementary material for: Patient satisfaction with computer-assisted structured initial assessment facilitating patient streaming to emergency departments and primary care practices: results from a cross-sectional observational study accompanying the DEMAND intervention in Germany
Source: BMC Prim Care. 2022 Aug 23;23:213. doi: 10.1186/s12875-022-01825-5 (PMC9397153; doi:10.1186/s12875-022-01825-5)
Supplement: Supplementary file 2 — Additional file 2. Patient questionnaire. [file 12875_2022_1825_MOESM2_ESM.docx]

**Patient questionnaire**

**Project „DEMAND – Personal initial assessment”**

**First of all, we would like to ask you a few questions about yourself.**

| **DAT1 Please specify the calendar date of today** |
| --- |
| ⎣ ⎦ ⎣ ⎦ . ⎣ ⎦ ⎣ ⎦ . ⎣ ⎦ ⎣ ⎦ ⎣ ⎦ ⎣ ⎦ |
|  |
| **SDD1 Month and year of birth** |
| ⎣ ⎦ ⎣ ⎦ . ⎣ ⎦ ⎣ ⎦ ⎣ ⎦ ⎣ ⎦ |
|  |
| **SDD2 Sex** |
| ⬜ female |
| ⬜ male |
| ⬜ diverse |
|  |
| **SDD3 At your home, do you live alone or together with other people? If you live with other people, who are they?** *(Multiple answers are permitted)* |
| ⬜ alone |
| ⬜ together with spouse |
| ⬜ together with child/children |
| ⬜ together with my parents or the parents of my spouse |
| ⬜ together with other family members |
| ⬜ together with other people (eg, friends or members of a shared flat) |
|  |
| **SDD4 What is your highest general school leaving certificate?** |
| ⬜ no school leaving certificate |
| ⬜ *Hauptschule* (9 school years) or 8-year *Grundschule* (former GDR before 1965) |
| ⬜ *Realschule* (10 school years) or *Polytechnische Oberschule* (10 school years) |
| ⬜ *Fachhochschulreife* (12 school years) |
| ⬜ *Abitur* (12 or 13 school years) |

| **SDD5 What is your highest vocational qualification?** |
| --- |
| ⬜ no vocational qualification |
| ⬜ *beruflich-betriebliche Ausbildung* (apprenticeship with vocational school) |
| ⬜ *beruflich-schulische Ausbildung* (vocational school) |
| ⬜ *Fachschule, Meisterschule, Technikerschule, Berufsakademie* or *Fachakademie* (vocational school) |
| ⬜ *Fachhochschule* (University of Applied Sciences) |
| ⬜ University |
|  |
| **SDD6 In which country were you born?** *(Please consider the national borders at that time)* |
| ⬜ In Germany |
| ⬜ In another country, please specify ⎣ ⎦ |
|  |
| **SDD7 In which country were your parents born?**  *(Please consider the national borders at that time)* |
| Mother  ⬜ In Germany  ⬜ In another country, please specify ⎣ ⎦ |
| Father  ⬜ In Germany  ⬜ In another country, please specify ⎣ ⎦ |

### With which health problem do you come to the hospital today?

*Please describe your health problem and the complaints because of which you are visiting the clinic today, for example: “I twisted my ankle, have pain in my foot and can no longer walk properly” or “I have a fever, cough, chest pain and feel limp ".*

*If pain or other complaints are named, please note where the pain / complaints occur.*

*Please write legibly, preferably in block capitals*

__________________________________________________________________________

__________________________________________________________________________

__________________________________________________________________________

__________________________________________________________________________

| **BES1 How urgently do you need medical treatment?**  *(Please tick the number that best describes the urgency of your treatment from your point of view.)* |
| --- |

| **No urgent treatment needed** | **0** | **1** | **2** | **3** | **4** | **5** | **6** | **7** | **8** | **9** | **10** | **Very urgent,  life threatening** |
| --- | --- | --- | --- | --- | --- | --- | --- | --- | --- | --- | --- | --- |
|  |  | | | | | | | | | | |  |

|  | | | | |
| --- | --- | --- | --- | --- |
| **BES2 How long have you had the complaints for which you came to the hospital?** | | | | |
| ⎣ ⎦ | | | | |
|  | | | | |
| **During your conversation today at the registration …** | **clearly no** | **rather no** | **rather yes** | **clearly yes** |
| **ZUF1 … did you feel that the staff member hat enough time for you?** | ⬜ | ⬜ | ⬜ | ⬜ |
| **ZUF2 … did he show interest in your personal situation?** | ⬜ | ⬜ | ⬜ | ⬜ |
| **ZUF3 Could you say everything that you wanted to say?** | ⬜ | ⬜ | ⬜ | ⬜ |
| **ZUF4 Would you recommend the hospital to friends with acute health problems?** | ⬜ | ⬜ | ⬜ | ⬜ |
